# Maxillofacial morphological factors related to acceleration of maxillary growth attributed to facial mask treatment: a structural superimposition study

**DOI:** 10.1186/s40510-018-0254-9

**Published:** 2019-01-14

**Authors:** Takashi S. Kajii, Yui Sakaguchi, Yoshihiko Sawa, Sachio Tamaoki

**Affiliations:** 1 Section of Orthodontics, Department of Oral Growth and Development, Fukuoka Dental College, 2-15-1 Tamura, Sawara-ku, Fukuoka, 814-0193 Japan; 2 Department of Oral Function & Anatomy, Okayama University Graduate School of Medicine, Dentistry and Pharmaceutical Sciences, Okayama, Japan

**Keywords:** Orthodontics, Orthopedic force, Facial mask, Maxillofacial sutures, Pterygoid fissure–palatine’s pyramidal process conjugation

## Abstract

**Background:**

Anatomical textbooks mention that the contact between the pterygoid process and the palatine’s pyramidal process is not a “suture” but “conjugation.”.The aim was to evaluate the maxillofacial morphological factor responding most to the orthopedic force of facial mask treatment, using the structural superimposition analysis.

**Methods:**

Thirty-one girls with Angle Class III malocclusion treated using a facial mask (FM group) and 11 girls with pseudo-Class III malocclusion (pseudo-III group) were examined. Lateral cephalograms at pre- and posttreatment were analyzed to evaluate maxillofacial changes. Cephalometric structural superimposition analysis originating with Björk and Skieller was also performed.

**Results:**

In the FM group, a multiple linear regression model showed that maxillary sutural growth was significantly associated with counter-clockwise rotation of the maxilla and treatment changes in the anteroposterior distance from the pterygomaxillary fissure to the maxillary anterior alveolus, not changes in the distance from the nasion to the maxillary anterior alveolus.

**Conclusions:**

Structural superimposition analysis showed that counter-clockwise rotation of the maxilla and changes in the distance from the pterygomaxillary fissure to the maxillary anterior alveolus responded most to the orthopedic force of facial mask treatment. The analysis implicated that the pterygoid fissure–palatine’s pyramidal process conjugation responds most to facial mask treatment among maxillofacial sutures and conjugation, and that the difference in the response induces maxillary counter-clockwise rotation.

**Electronic supplementary material:**

The online version of this article (10.1186/s40510-018-0254-9) contains supplementary material, which is available to authorized users.

## Background

In the mixed dentition, the treatment plan for a patient diagnosed with Angle Class III malocclusion with a retrognathic maxilla aims to improve intermaxillary relationships using maxillary protraction appliances [1–8]. Many studies have reported the effects of maxillary protraction appliances, especially facial mask, on maxillary growth and mandibular clockwise rotation [3, 5–10].

Sutural growth, endochondral growth of the nasal septum, and periosteal growth of cortical bone are included in maxillary growth. Animal studies and a finite element study using dry skulls have suggested sutural modification as the most important determinant of sagittal growth in the naso-maxillary complex [11–13]. However, conventional cephalometric analysis cannot evaluate the effects on maxillary growth by separating sutural growth and changes in maxillary morphology derived from periosteal growth, because external anatomical points of the maxilla (e.g., point A, anterior nasal spine (ANS)) depend on local surface remodeling processes and may lead to erroneous assessment regarding maxillary growth [14]. Using a structural (or regional) superimposition analysis [15] based on Björk and Skieller [16, 17], Sakaguchi et al. [18] first assessed the effects of facial mask on maxillary and mandibular growth by separating maxillary sutural growth, mandibular condylar growth, and mandibular total rotation. The study [18] suggested that accelerated maxillary sutural growth and inhibited counter-clockwise total rotation of mandibular corpus growth attributed to facial mask treatment may contribute to improvements in Angle Class III malocclusion.

Animal studies [11, 12] suggested that the zygomaticomaxillary, zygomaticotemporal, frontomaxillary, zygomaticofrontal, transverse palatine, and sphenozygomatic sutures, and the pterygoid fissure–palatine’s pyramidal process conjugation (not suture) [19] respond histologically to the orthopedic force of facial mask. However, it is not clear which sutures and conjugations respond most to the orthopedic force of facial mask. Structural superimposition analysis may facilitate a better understanding of which sutural growth is most accelerated by facial mask treatment.

The purpose of the present retrospective study was to evaluate maxillofacial morphological factors responding most to the orthopedic force of facial mask treatment, using structural superimposition analysis.

## Methods

### Subjects

The ethics committee of the institution of the author’s affiliation approved all protocols in this retrospective study (approval no. 214). Subjects gave written informed consent for participation in this study.

The subjects consisted of 31 Japanese girls with Angle Class III malocclusion treated using a facial mask (FM group). Criteria for including an Angle Class III patient in the study were (1) overjet ≤ 0.0 mm; (2) Wits appraisal <− 2.0 mm; (3) Class III molar relationships; (4) retrognathic maxilla (point A to nasion perpendicular < 0.5 mm); and (5) age ≥ 6 years and ≤ 9 years at initial examination. Criteria for excluding a subject from the study were (1) presence of congenital anomalies, (2) history of trauma, and (3) previous orthodontic treatment.

The designs of the extra-oral facial mask and intra-oral appliance for the subjects were described in the previous study [18]. Briefly, bands were fitted on the maxillary permanent first molars or primary first molars for the intra-oral appliance. The extra-oral facial mask was a one-piece construction with an adjustable anterior wire and hooks. To avoid bite opening during repositioning of the maxilla, protraction elastics were attached near the maxillary canines with downward and forward pull of 30° to the occlusal plane. Mean treatment duration was 14 ± 4 months. Standardized lateral cephalograms were obtained before (T1; mean age, 8.0 ± 1.4 years) and after (T2; mean age, 9.2 ± 1.4 years) facial mask treatment from all subjects in the FM group.

Pseudo-Class III malocclusion is characterized by an anterior crossbite caused by a functional forward position of the mandible [7]. Another subjects consisted of 11 Japanese girls with pseudo-Class III malocclusion (pseudo-III group) were also examined. Inclusion and exclusion criteria for potential subjects in the pseudo-III group were the same as described in the previous study [18]. All subjects wore a lingual arch with spring [20] for improvement of the anterior crossbite by inclining the maxillary incisors labially. No subjects were treated using an orthopedic appliance. Standardized lateral cephalograms were obtained before treatment (T1; mean age, 8.1 ± 0.9 years) and during growth observation (T2; mean age, 9.4 ± 0.9 years) from all subjects in the pseudo-III group. Mean ages at T1 and T2 were almost the same for the FM and pseudo-III groups.

For all vectors and cephalometric measurements with significant differences in values between the FM and pseudo-III groups, the appropriate sample size was estimated at 6.18–26.11 (α (significance level of type I error) = 0.05, ß (significance level of type II error) = 0.20). The number of subjects in the FM and pseudo-III groups was 31 and 11, respectively, and was almost consistent with the proper sample size.

### Measurements and assessments

In addition to conventional cephalometric variables, some linear measurements were performed by projecting landmarks on the vertical lines perpendicular to the Frankfurt Horizontal (FH) plane through the sella (S), the pterygomaxillary fissure (Ptm), or nasion (N). The cephalometric measurements used in the present study were the same as described in the previous study [18].

Cephalometric analysis derived from the original analysis of Björk and Skieller [16] was performed to evaluate facial mask effects by decomposing T1-T2 changes into maxillary skeletal growth and maxillary molar movement. The structural superimposition method for this analysis was applied as described previously [16, 18] and is briefly explained below.

**Fig. 1:**
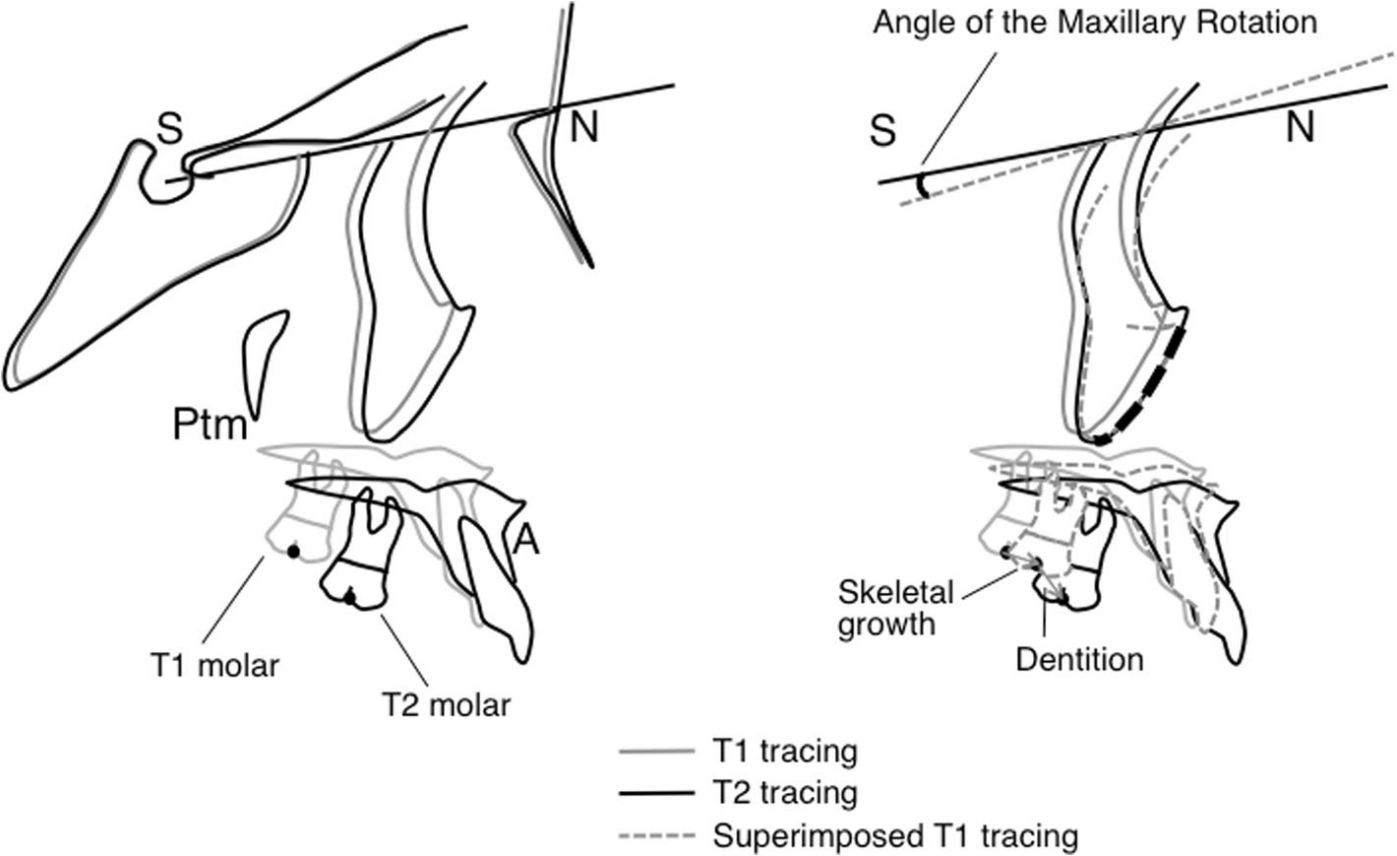
Left: Superimposition of pre-treatment (initial, T1) and posttreatment (final, T2) tracings on the cranial base. Right: Superimposition of T1 and T2 tracings on the maxillary internal stable structure, the anterior contour of the zygomatic processes (shown by a black broken line). The maxillary dental component and maxillary skeletal component are represented. The maxillary rotation was measured as the angle between the sella-nasion (SN) lines of T1 and T2 tracings on the superimposition. Ptm: the pterygomaxillary fissure; A: point A

Figure 1 shows superimposition of the T1 and T2 tracings on the maxillary internal stable structure, the anterior contours of the zygomatic processes. Tracing and superimpositions were performed manually. “T1 tracing” was superimposed on the cranial base of the T2 tracing. “Superimposed T1 tracing” was superimposed on the maxillary internal stable structure of the T2 tracing. The dental component was represented as the distance between measurement points of the molar on the T2 tracing and the superimposed T1 molar on the T2 tracing. The skeletal component was represented as the distance between measurement points of the molar on the T1 tracing and the superimposed T1 molar on the T2 tracing. The amount and direction of rotation of the maxilla (maxillary rotation) were measured as the angle between the sella-nasion (SN) lines of the superimposed T1 tracing and the T2 tracing on superimposition. A positive value for maxillary rotation means counter-clockwise (backward) rotation of the maxilla.

**Fig. 2:**
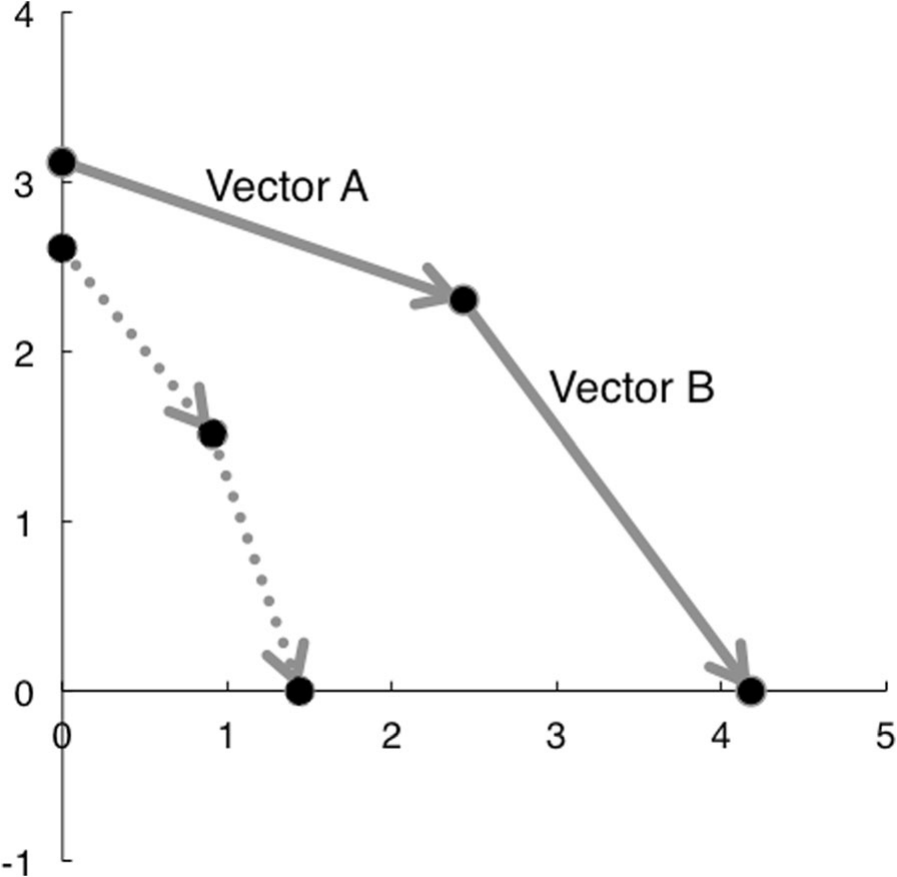
Superimposed graphic vector representations of maxillary molars in the FM and pseudo-III groups. Solid line: FM group; broken line: pseudo-III group. Vector A: maxillary skeletal growth component; vector B: maxillary dental component. The functional occlusal plane, a line drawn through the occlusal surfaces of the maxillary and mandibular first molars and first and second deciduous molars, of the T2 cephalogram was used as the *x*-axis for the sagittal direction

As a result of the structural superimposition method, two components were obtained: vector A, vector maxillary skeletal growth; and vector B, vector maxillary dentition [18]. Figure 2 shows graphical vector presentations of movement of the maxillary molar. The functional occlusal plane, a line drawn through the occlusal surfaces of the maxillary and mandibular first molars and the first and second primary molars, of the T2 cephalogram was used as an *x*-axis for the sagittal direction. The *y*-axis was perpendicular to the functional occlusal plane through a measurement point of the molar of the T1 tracing. The *x*- and *y*-components of vectors A and B were measured based on this coordinate system. Vector B was not evaluated in the present study because the vector showed maxillary dentition.

### Statistical methods

To determine the reproducibility of this method, ten subjects were randomly selected. The superimpositional measurements and all angular and linear measurements at T1 and T2 were repeated at least 4 weeks after the first measurements. The combined error (*S*_e_) and coefficient of reliability were calculated according to Houston [21]. Combined error (*S*_e_) was estimated by the formula *S*_e_^2^ = ∑d^2^/2n, where *d* is the difference between the first and second measurements, and *n* is the sample size. The coefficient of reliability was estimated by the formula 1-*S*_e_^2^/*S*_t_^2^, where *S*_t_ is the total variance of the measurement. The coefficient of reliability was greater than 90% and was considered to be within acceptable limits for all measurements.

Vector A, the *y*-component of vector A, and all cephalometric measurements were normally distributed, except the *x*-component of vector A. However, the number of the pseudo-III group (*n* = 11) was too small to insure the possibility of normal distribution. The Mann-Whitney *U* test was thus used to compare vector A, the *x*- and *y*-components of vector A, and maxillary rotation from the structural superimposition analysis between the FM and pseudo-III groups. Multiple linear regression analysis was used to identify associations between vector A and T1-T2 changes in cephalometric angular or linear measurement values including maxillary rotation in the FM group. Vector A and other values were used as dependent and independent variables, respectively. Stepwise variable selection was used to identify good associations of dependent variables to independent variables. Multiple linear regression analysis was not used in the pseudo-III group because the number of the group was only 11. Statistical analyses were performed using SPSS version 23.0 statistical package (SPSS, Chicago, IL). The level of significance was set at *P* < 0.05.

## Results

Table 1 shows the means and standard deviations of structural superimpositional measurements of the FM and pseudo-III groups. The FM group had significantly larger vector A (maxillary sutural growth) (*P* < 0.001) and *x*-component of vector A (*P* < 0.001) as compared to the pseudo-III group. The FM group had significantly larger counter-clockwise maxillary rotation (*P* < 0.01) compared to the pseudo-III group. Figure 2 shows superimposed graphic vector representations of the maxilla for the two groups. The FM group exhibited a longer vector A than the pseudo-III group, so the maxillary molar was found to move more anteriorly in the FM group.

**Table 1:** Structural superimpositional measurements in the FM and pseudo-III groups

	FM			Pseudo-III			*P* value
	Median	Mean	SD	Median	Mean	SD	
Maxillary sutural							
Vector A (mm)	2.69	2.78	1.28	1.60	1.54	0.64	0.005**
Vector A (x) (mm)	2.34	2.52	1.40	0.77	0.91	0.63	< 0.001***
Vector A (y) (mm)	− 0.69	− 0.83	0.64	− 1.29	− 1.10	0.61	0.131
Maxillary rotation (°)	2.00	1.96	1.30	0.50	0.69	0.90	0.003**

*FM* facial mask, *pseudo*-*III* pseudo-Class III malocclusion, *SD* standard deviation

** *P*<0.01, *** *P*<0.001

Tables 2 and 3 show multiple linear regression models of the influence on vector A and the *x*-component of vector A, respectively. In the FM group, vector A and the *x*-component of vector A were significantly associated with maxillary rotation and T1-T2 changes in Ptm’-A’ (the distance between point A and Ptm projected perpendicularly on the line parallel to the FH plane through the point A), whereas vector A and the *x*-component of vector A were not significantly associated with maxillary rotation and T1-T2 changes in Ptm’-A’ in the pseudo-III group (Pearson’s correlation coefficient) (data not shown). Multiple regression models of the FM group offered prediction capabilities of about 64% (*R*^2^ = 0.643, *P* < 0.001) for vector A and about 52% (*R*^2^ = 0.516, *P* < 0.001) for the *x*-component of vector A.

**Table 2:** Multiple linear regression analysis of the influence on vector A in the FM group

Model	Coefficient			Correlations
	B	t	*P*	Partial
Constant	0.866	2.802	0.009	
Maxillary rotation	0.562	4.739	< 0.001	0.710
Ptm’-A’	0.547	3.286	0.003	0.597

*R* = 0.802, *R*^2^ = 0.643, *P* < 0.001

*FM* facial mask, *pseudo*-*III* pseudo-Class III malocclusion, *Ptm*’-*A*’ the distance between point A and Ptm (the pterygomaxillary fissure) projected perpendicularly on the line parallel to the FH plane through the point A, *PUFH* posterior upper facial height

**Table 3:** Multiple linear regression analysis of the influence on the *x*-component of vector A in the FM group

Model	Coefficient			Correlations
	B	*t*	*P*	Partial
Constant	0.643	1.639	0.112	
Maxillary rotation	0.539	3.581	0.001	0.631
Ptm’-A’	0.552	2.615	0.014	0.543

*R* = 0.719, *R*^2^ = 0.516, *P* < 0.001

*FM* facial mask, *pseudo*-*III* pseudo-Class III malocclusion, *Ptm*’-*A*’ the distance between Point A and Ptm (the pterygomaxillary fissure) projected perpendicularly on the line parallel to the FH plane through point A, *PUFH* posterior upper facial height

In regards to the *y*-component of vector A, T1-T2 changes in the FH plane to palatal plane angle only showed a significant correlation with the *y*-component of vector A in the FM group (Pearson’s correlation coefficient). No value of changes in cephalometric angular or linear measurements including the maxillary rotation showed a significant correlation with the *y*-component of vector A in the pseudo-III group. Therefore, multiple linear regression models were not constructed for the *y*-component of vector A.

## Discussion

Counter-clockwise rotation of the maxilla significantly influenced a large amount of the maxillary sutural growth during facial mask treatment in the present study (Tables 2 and 3). Kambara [11] showed that significant morphological and histological changes in the circum-maxillary sutures caused anterior displacement and slight counter-clockwise rotation of the maxillary complex in a study of maxillary protraction appliances on *Macaca irus* monkeys. Nanda and Hickory [12] also showed that the direction of the histologic response of the zygomaticomaxillary suture was related to the location of the center of rotation in a study of maxillary protraction appliances in *M*. *irus* monkeys. Ishii et al. [1] reported that the maxilla was displaced more anteriorly and rotated more counter-clockwise with first molar protraction than with first premolar protraction in a study of maxillary protraction appliances in orthodontic patients. The present results could confirm that counter-clockwise rotation of the maxilla, which correlates with the sagittal component of maxillary sutural growth, contributes to the forward position of the maxilla attributed to facial mask treatment for orthodontic patients.

Treatment changes in Ptm’-A’, the distance from the pterygomaxillary fissure to the maxillary anterior alveolus, were also significantly correlated with a large amount of the maxillary sutural growth during facial mask treatment in the present study (Tables 2 and 3). The pterygomaxillary fissure is located under the pterygopalatine fossa that shows inverted teardrop-shaped radiolucency. The reason why no changes in other cephalometric measurements for assessment of the anteroposterior position of the maxilla (such as the SNA angle and point A to nasion perpendicular) but changes in the distance from the pterygomaxillary fissure to the maxillary anterior alveolus were significantly correlated with maxillary sutural growth is described below.

In many histological and clinical studies [11, 12, 22] of maxillofacial growth and orthopedic treatment, the contact between the pterygoid process and the pyramidal process of the palatine (or the maxillary tuberosity) was considered to be the pterygopalatine (or pterygomaxilla) “suture.” Sutures are limited to the skull and occur wherever margins or broader surfaces of bones are separated only by connective tissue, the sutural ligament or membrane, which is a surviving unossified part of mesenchymatous sheets in which dermal bones grow. However, in anatomical textbooks [19, 23], the medial and lateral plates of the pterygoid process are separated below by the angular pterygoid fissure, whose margins “articulate” with the palatine’s pyramidal process. In other words, the pyramidal process of the palatine slopes down into the angular pterygoid fissure. Thus, contact between the pterygoid process and the pyramidal process of the palatine is not a “suture,” but “conjugation.” In fact, pubertal dry skulls show that the pterygoid fissure–palatine’s pyramidal process conjugation does not result in contact. A space or gap is found at the conjugation.

Angelieri et al. [24, 25] reported that the early maturational stages of the zygomaticomaxillary suture were associated directly with a greater response to a facial mask. Bone conjugation is thus thought to respond more easily to orthopedic force than the bone sutures. In the maxillofacial sutures and bone conjugation, the pterygoid fissure–palatine’s pyramidal process conjugation may separate easiest with facial mask treatment. The anatomical viewpoint may be the reason why changes in the distance from the pterygomaxillary fissure to the maxillary anterior alveolus, not changes in other cephalometric measurements for assessment of the anteroposterior position of the maxilla, were significantly correlated with maxillary sutural growth during facial mask treatment. Furthermore, this viewpoint suggests that the pterygoid fissure–palatine’s pyramidal process conjugation responds most to the orthopedic force of facial mask treatment among the maxillofacial sutures and bone conjugations.

Some studies [1, 12] of maxillary protraction appliance suggested that the center of rotation of the maxilla is located just above the frontomaxillary suture. The pterygoid fissure–palatine’s pyramidal process conjugation is farther from the location of the center of rotation than the zygomaticomaxillary and zygomaticofrontal sutures and the frontomaxillary suture (close to the nasion). These anatomical locations may also be the reason why changes in the distance from the pterygomaxillary fissure to the maxillary anterior alveolus, not changes in other cephalometric measurements (such as the SNA angle and point A to nasion perpendicular) for assessment of the anteroposterior position of the maxilla, were significantly correlated with maxillary sutural growth during facial mask treatment. In addition, the locations may be the reason why counter-clockwise rotation of the maxilla significantly influenced a large amount of the maxillary sutural growth during facial mask treatment.

The limitation in the present study lies in invisualization of the pterygoid fissure–palatine’s pyramidal process conjugation in two-dimensional lateral cephalograms. To evaluate which maxillofacial sutures and bone conjugations respond most to orthopedic force, histological, computed tomographic, or finite element study would be better. However, to evaluate the response in patients, it would not be easy to obtain the large amount of data for these studies. In the future, we will try thin plate spline analysis using lateral cephalogram for three-dimensional evaluation. The limitation in the present study lies also in sample size to allow multiple liner regression analysis. The sample size is generally over ten times the number of variables [26], although there are many articles those used only 16–40 samples to allow multiple liner regression analysis with more than 4 variables [27–30].

From a clinical point of view, orthodontists should recognize that the expected effects of facial mask treatment on maxillary growth might differ between patients with short- and long-face, because the present results suggest more favorable effects on maxillary sutural growth are accompanied by a larger amount of counter-clockwise rotation of the maxilla. The larger amount of counter-clockwise rotation of the maxilla would induce mandibular clockwise rotation and a subsequent increment of anterior facial height.

Long-term effects (at the end of growth) on maxillary growth in Class III patients with facial mask treatment remain unclear. The structural superimposition method may allow clarification of questions of long-term effects, because the method can decompose maxillary growth into maxillary skeletal growth.

## Conclusions

Structural superimposition analysis showed that the greater the acceleration of maxillary sutural growth due to facial mask treatment, the greater the increase in the anteroposterior distance from the pterygomaxillary fissure to the maxillary anterior alveolus. Structural superimposition analysis therefore implicated that the pterygoid fissure–palatine’s pyramidal process “conjugation” responds most to the orthopedic force of facial mask treatment among maxillofacial “sutures” and “bone conjugations,” and that the difference in the response induces counter-clockwise rotation of the maxilla.

## Additional file

Additional file 1: All datasets. (XLS 55 kb)

## References

[1] Ishii H, Morita S, Takeuchi Y, Nakamura S (1987). Treatment effect of combined maxillary protraction and chincap appliance in severe skeletal class III cases. Am J Orthod Dentofac Orthop.

[2] Baccetti T, Franchi L, McNamara JA, Brudon WL (2001). Class III malocclusion. Orthodontics and dentofacial orthopedics.

[3] Franchi L, Baccetti T, McNamara JA, Brudon WL (2001). The facial mask. Orthodontics and dentofacial orthopedics. Ann Arbor: Needham press Inc.

[4] Yoshida I, Shoji T, Mizoguchi I (2007). Effects of treatment with a combined maxillary protraction and chincap appliance in skeletal class III patients with different vertical skeletal morphologies. Eur J Orthod.

[5] De Toffol L, Baccetti T, Franchi L, Cozza P (2008). Orthopedic treatment outcomes in class III malocclusion. A systematic review Angle Orthod.

[6] Fudalej P, Dragon M, Wedrychowska-Szulc B (2011). Prediction of the outcome of orthodontic treatment of class III malocclusions—a systematic review. Eur J Orthod.

[7] Ngan P, Moon W (2015). Evolution of class III treatment in orthodontics. Am J Orthod Dentofac Orthop.

[8] De Clerck HJ, Proffit WR (2015). Growth modification of the face: a current perspective with emphasis on class III treatment. Am J Orthod Dentofac Orthop.

[9] McNamara JA (1987). An orthopedic approach to the treatment of class III malocclusion in young patients. J Clin Orthod.

[10] Delaire J (1997). Maxillary development revisited: relevance to the orthopaedic treatment of class III malocclusions. Eur J Orthod.

[11] Kambara T (1977). Dentofacial changes produced by extraoral forward force in the Macaca irus. Am J Orthod.

[12] Nanda R, Hickory W (1984). Zygomaticomaxillary suture adaptations incident to anteriorly-directed forces in rhesus monkeys. Angle Orthod.

[13] Miyasaka-Hiraga J, Tanne K, Nakamura S (1994). Finite element analysis for stresses in the craniofacial sutures produced by maxillary protraction forces applied at the upper canines. Br J Orthod.

[14] Halazonetis DJ (1998). Cephalometric analysis of changes in occlusal relationship. Eur J Orthod.

[15] Haralabakis NB, Halazonetis DJ, Sifakakis IB (2003). Activator versus cervical headgear: superimpositional cephalometric comparison. Am J Orthod Dentofac Orthop.

[16] Björk A, Skieller V (1977). Roentgencephalometric growth analysis of the maxilla. Trans Eur Orthod Soc.

[17] Björk A, Skieller V (1983). Normal and abnormal growth of the mandible. A synthesis of longitudinal cephalometric implant studies over a period of 25 years. Eur J Orthod.

[18] Sakaguchi Y, Kajii TS, Kumano C, Tamaoki S, Ishikawa H (2017). Effects of facial mask treatment are attributed to accelerated maxillary growth and inhibited counter-clockwise total rotation of the mandibular corpus: a structural superimposition study. Orthod Waves.

[19] Soames RW, Williams PL (1995). Skeletal system. Gray’s anatomy. 38th ed.

[20] Daskalogiannakis J (2000). Glossary of orthodontic terms.

[21] Houston WJ (1983). The analysis of errors in orthodontic measurements. Am J Orthod.

[22] Ross RB (1970). The clinical implications of facial growth in cleft lip and palate. Cleft Palate J.

[23] Norton NS, Norton NS (2007). Osteology. Netter’s head and neck anatomy for dentistry.

[24] Angelieri F, Franchi L, Cevidanes LHS, Hino CT, Nguyen T, McNamara JAJ (2017). Zygomaticomaxillary suture maturation: a predictor of maxillary protraction? Part I - A classification method. Orthod Craniofac Res.

[25] Angelieri F, Ruellas AC, Yatabe MS, Cevidanes LHS, Franchi L, Toyama-Hino C (2017). Zygomaticomaxillary suture maturation: part II—the influence of sutural maturation on the response to maxillary protraction. Orthod Craniofac Res..

[26] Peduzzi P, Concato J, Feinstein AR, Holford TR (1995). Importance of events per independent variable in proportional hazards regression analysis II. Accuracy and precision of regression estimates. J Clin Epidemiol.

[27] Kajii TS, Fujita T, Sakaguchi Y, Shimada K. Osseous changes of the condyle affect backward-rotation of the mandibular ramus in angle class II orthodontic patients with osteoarthritis of the temporomandibular joint. Cranio. 2018; 10.1080/08869634.2017.1421446. 10.1080/08869634.2017.1421446 29359644

[28] Radwan ES, Montasser MA, Maher A (2018). Influence of geometric design characteristics on primary stability of orthodontic miniscrews. J Orofac Orthop.

[29] Hamdan AL, Khandakji M, Macari AT (2018). Maxillary arch dimensions associated with acoustic parameters in prepubertal children. Angle Orthod..

[30] Albogha MH, Takahashi I. Effect of loaded orthodontic miniscrew implant on compressive stresses in adjacent periodontal ligament. Angle Orthod. 2018; 10.2319/122017-873.1. 10.2319/122017-873.1 PMC8120879 30230377

